# Insect Pests and Integrated Pest Management in Museums, Libraries and Historic Buildings

**DOI:** 10.3390/insects6020595

**Published:** 2015-06-16

**Authors:** Pascal Querner

**Affiliations:** 1 Department of Integrated Biology and Biodiversity Research, Institute of Zoology, University of Natural Resources and Life Sciences, Gregor-Mendel-Straße 33, Vienna A-1180, Austria; E-Mail: pascal.querner@boku.ac.at; 2 Institute of Archäometrie, Universitity of Applied Arts Vienna, Expositur Salzgries, Vienna A-1010, Austria

**Keywords:** IPM, museums, prevention, insect pests

## Abstract

Insect pests are responsible for substantial damage to museum objects, historic books and in buildings like palaces or historic houses. Different wood boring beetles (*Anobium punctatum*, *Hylotrupes bajulus*, *Lyctus* sp. or introduced species), the biscuit beetle (*Stegobium paniceum*), the cigarette beetle (*Lasioderma serricorne*), different Dermestides (*Attagenus* sp., *Anthrenus* sp., *Dermestes* sp., *Trogoderma* sp.), moths like the webbing clothes moth (*Tineola bisselliella)*, Silverfish (*Lepisma saccharina*) and booklice (*Psocoptera)* can damage materials, objects or building parts. They are the most common pests found in collections in central Europe, but most of them are distributed all over the world. In tropical countries, termites, cockroaches and other insect pests are also found and result in even higher damage of wood and paper or are a commune annoyance in buildings. In this short review, an introduction to Integrated Pest Management (IPM) in museums is given, the most valuable collections, preventive measures, monitoring in museums, staff responsible for the IPM and chemical free treatment methods are described. In the second part of the paper, the most important insect pests occurring in museums, archives, libraries and historic buildings in central Europe are discussed with a description of the materials and object types that are mostly infested and damaged. Some information on their phenology and biology are highlighted as they can be used in the IPM concept against them.

## 1. Introduction

### 1.1. IPM in Museums

Integrated Pest Management (IPM) in museums, libraries, archives and historic buildings is an important part of preventive conservation, focusing on the prevention of pest infestations and the reduction of pesticide application. The concept of IPM was developed in the 1950s in the food industry. Starting in the 1980s, it has also been applied in museums [1,2,3], mainly in the UK, USA, Canada and Australia. Today it is applied in more and more museums, including also smaller collections, regional museum in the countryside and also in tropical countries. Pest Management strategies for museums were described by [4,5,6,7,8,9,10,11]. The most comprehensive books on IPM in museums are by [12,13,14,15]. Another important source of information for people working with IPM are the regular international conferences (see for example [16,17,18]) and the homepage www.museumpests.net [19].

In the past application, pesticides were used to treat infestation when they were obvious, but the source of the infestation was often not found. It was also not always 100% certain that the insects found were pests damaging objects, as the correct identification was not part of the pest management concept. Today, most museums lack documentation when and what types of pesticides (mainly insecticides, but also fungicides) were used in the history of the collection. Since the IPM concept is applied, treatment is only a part of the concept, and a lot of resources are spend on prevention and monitoring.

To prevent damage by pests and their introduction, a holistic concept is applied: This is achieved by sealing the building against pest entry, adapting the micro-climate (the cooler the indoor climate, the slower they develop and reproduce), maintaining high hygienic standards (cleaning is an important part of IPM to reduce food sources for pests), quarantining all new and incoming objects and monitoring pest infestations with traps. Further staff training is an important part of IPM. If an active infestation is found, objects are isolated as fast as possible and non-chemical methods like freezing, heating or anoxia treatments are preferred to prevent damage to the objects or harm museum staff.

### 1.2. Most Vulnerable Collections in Museums

Insects infest not all collections of objects in an equal ways. Mostly natural history collections with large numbers of dried insects, usually stored in drawers, dry plant material in herbaria, stuffed animals, fur and skeleton specimens are at a very high risk of infestation and damage. Large numbers of objects of these vulnerable materials (very attractive as food for some insects) are stored close to each other and in dark areas (stores). This helps the spread of an infestation from one drawer or closet to the other. 

The second high-risk collections are ethnographic objects, which have similar materials as the natural history museums. In addition, a large amount of fur, feather, leather, plant materials or wood are stored together. Many of these objects like pumpkin vessels or textiles are stained (with food, body oil, sweat or urine), which is part of their use and cultural history and make them even more attractive for the insects feeding on them.

Historic and modern art museums also suffer from insect damage, but here it depends very much on the materials. Most historic furniture has some exit holes by *Anobium punctatum* (or other species) and also historic textiles often have some damage by moths or beetles. However, if art collections are well looked after, they can stay without pests for long periods of time.

Libraries and archives are also collections, where large numbers of very similar materials are very close together. Only a few insect species are feeding on paper and the historic bookbinding, but they can result in severe damage if the infestation is not found.

Historic buildings like castles, palaces, or old museum buildings usually have resident populations of insect pests found in shafts, unused chimneys, under wooden floors or behind wooden walls. Finding and getting rid of these pest populations is often very difficult and costly. Here, the prevention of the spread of these populations to historic objects or the buildings themselves is an important part of the IPM.

### 1.3. Monitoring of Insects in Museums

Insect pest monitoring is an important part in IPM to detect active infestations. Collecting specimens helps to correctly identify the species involved and to locate the infested objects or problems within the building [20,21,22,23,24,25,26]. All these aspects require a trained person: This person coordinates the IPM project, collection of data, coordination of treatments and sets priorities for further actions.

Mostly sticky blunder traps and pheromone lures (for webbing clothes moths for example) are used to collect the required data for the monitoring. Traps and results are visualised on floor maps of the building, which helps to find infested objects or locate problems related to the building. In addition, bait traps (larval food monitoring) and UV light traps are used to monitor insect activity. Visual inspections are also an important way to collect data on the activity of pests.

### 1.4. Staff Responsible for the IPM in Large Collections

Few museum organisations are big enough to fund a fulltime position for IPM; exceptions are, for example, English Heritage in the UK, where currently IPM is installed in 62 sites [27], the British Museum in London [28], the Victoria & Albert Museum in London [29], the Natural History Museum of London [30], Colonial Williamsburg [31] or the National Museums of Berlin, Stiftung Preußischer Kulturbesitz [32]. If no fulltime position for IPM is present in a collection, the IPM work is usually done by the conservationists or other museum staff responsible for the collections. Treatments are often done by the museum staff themselves using household chest freezers, heating chambers, nitrogen chambers or by using small anoxia bags (see information’s below). Sometimes, external companies are also responsible for treatments and/or monitoring. Here, a few very specialized companies have adapted their work to the requirements of the museums (high value of objects for example).

### 1.5. Treatments against Insect Pests

#### 1.5.1. Chemical Methods

In the past, similar as in the food industry, chemical methods were the preferred to treat active infestations. Today, after prohibition of the use of DDT, Methyl bromide and Hydrogen cyanide, few museums in Europe still regularly use pesticides against insect pests. Pyrethroid fumigations are not preferred, as they don’t kill all insect stages (for example, inside of the objects). One hundred percent success in killing at all stages can be achieved with toxic gases, but also these have their limitations to be used in museums. Phosphine, for example, cannot be used because of potential corrosion with metals like gold and silver. Sulfuryl fluoride is the only toxic gas used in museums from time to time, but it is costly and the restriction to use it within cities and close to apartments also limits its application. CO_2_, another chemical biocide, is sometimes applied in museums, and it is not harmful for the objects. Most museums will try to prevent the use of chemicals in their collections and refer to the non-chemical methods listed below. Sometimes Sulfuryl fluoride is used, for example, when no other method is available.

#### 1.5.2. Non-Chemical Methods

Insect pests (all stages) can be killed with different non-chemical methods and these methods are preferred in museum, libraries and historic buildings, as they don’t damage the objects, kill at all stages, and don’t harm the environment or health of museum staff. Physical treatments are achieved due to freezing [33,34,35], controlled heating [36,37], microwave radiation [38] or gamma radiation of the objects. However, not all materials and objects can be treated with these methods, for more delicate objects and mixed materials anoxic treatments are preferred [39,40,41,42]. Low oxygen atmosphere is achieved using Nitrogen, Argon or anoxia treatments with oxygen scavengers in small bags. Still quite new and under development is the biological method application using parasitoid wasps, for example, against biscuit beetles, webbing clothes moths, or furniture beetles [43,44,45,46,47,48]. First, results show that it is working if the location of the infestation is known and parasitoid wasps are commercially available (this is not the case for all pests, but just a small selection). See also Querner and Kjerulff [49] for an overview of treatment methods used in museums.

No single treatment method is perfect and the best method applied has to be selected depending on the time (last treatment from 24 h with heat to five weeks with nitrogen), financial recourses, availability and type of pests and materials to treat.

## 2. Insect Pests Found in Museums

### 2.1. Insect Pests on Wood

Different types of wood are stored in museums and can be infested by species of beetles feeding on structural timber, furniture, wooden floors, material used for displays, firewood, wooden pallets or other wooden objects. Most species prefer fresh softwood of high sapwood content rich in nutrients and also prefer high wood moisture. The wood being infested can vary between the species, which can help in the identification and prevention.

The most commune wood pest is probably the furniture beetle/woodworm (*Anobium punctatum)*, or at least it was until heating was installed in most buildings. The species needs a high wood moisture (+16%), which is usually not adequate for storing museum objects. It infests sapwood made of hardwood, ply with animal glue, some composite cellulose material and books. The second species needing a high wood moisture (hardwood) and also some fungi activity on the damp wood is the death watch beetle (*Xestobium rufovillosum*). It is seldom found inside of buildings and infesting often outdoor structures like open air museums, where climate control is not possible. Further species belonging to the same family Anobiidae are *Nicobium castaneum*, *Oligomerus ptilinoides* and *Ptilinus pectinicornis* and the Bostrichidae *Bostrichus capucinus*, all can be found in museums or historic buildings [50,51,52,53,54,55]. These species develop faster then *Anobium punctatum* (2–3 years under normal conditions) and are less dependent on high wood moisture. They occur more in the south of Europe and might be species profiting from climate change and heating inside buildings.

Wood feeding weevils are only found on very damp wood (but can feed also in ideal conditions on paper and books) like the New Zeeland wood weevil (*Euophryum confine)*, the pit-prop beetle (Hexarthrum exiguum) and *Pentarthrum huttoni.* In the infested catacombs in the Michaela crypt in Vienna the larvae of the *Pantarthrum huttoni* beetles were not only feeding on the wood coffins but also the mummies [56,57]. Because weevils can only feed on very damp wood, active infestations can be stopped by lowering the humidity inside the building from over 90% RH to below 60%.

Longhorn beetle *Hylotrupes bajulus* only infest softwood and are found as pests in attics or also wooden pallets. They don’t need a high wood moisture and are easy to detect as the adults leave the wood through large oval exit holes.

Powder post beetle on the contrary feed only on hardwood, and here also only on the sapwood. The most important species (some are introduced and only occur in buildings) are *Lyctus brunneus*, *Lyctus linearis*, *Lyctus cavicollis* and *Lyctus planicollis*. Because of their fast development (one year) damage can be very severe within a short time. Recently, a rise of problems in museums have developed with new wood being introduced into the museum, for example as picture frames, wooden floors or works of modern art, all of which were infested and resulted in costly treatments [38].

The species of wood boring beetles can result in large damages to museum objects. But because of their slow development (years; except for the *Lyctus* species), the infestation needs to be overlooked for many years. Most of the time, the larvae of the wood boring beetles are transported with infested objects into the collection. For many species, regulating the humidity is already a very effective way to stop or slow down the development.

### 2.2. Insect Pests on Keratine and Chitin Based Materials

A big group of insect pests found in museums are keratine feeding insects. Outside of buildings, they feed on dead animals, or live in nests of vertebrate or bird nests. Inside of museums, they feed on fur, feathers, animal skin, hair, bristles, animal wool, felt, silk, yarn, velvet, carpeting, insect specimens, parchment and vellum or stuffed animals. The most important species are fur beetles (*Attagenus*) like the black carpet beetle (*Attagenus unicolor*), the brown carpet beetle (*A. smirnovi*) and the two-spotted carpet beetle (*A. pellio*). In Austria, *A. unicolor* is the most wide spread; further to the north, it is being replaced by *A. smirnovi*, which seems to be more competitive in places with both species occurring [58,59]. *A. pellio* is usually found only in few individuals and was found damaging rhinoceros horn objects in a fine arts collection. Diverse groups of Dermestid beetles found in museums are carpet beetles, the most common being the varied carpet beetle *Anthrenus verbasci*. Less often and mostly only found as a few individuals in museums are the common carpet beetle (*A. scophiularidae)*, the museum beetle (*A. museorum*), the furniture carpet beetle (*A. flavipes*), the Guernsey carpet beetle (*A. sarnicus*), the carpet beetle (*Anthrenus pimpinellae*) or the Australian carpet beetle (*Anthrenocerus australis*). These species are responsible for damaging many dried insect collections, often resulting in complete loss of the insect specimen. They are very tolerant and can access collections and drawers through very small cracks, when the larvae are still very small. 

All these species prefer animal food, and a source of infestation can be animal nests or dead birds in a chimney, but they can feed also on dried plants, seeds, grains, dried milk and cereals.

The hide beetle *(Dermestes maculatus*), the larder beetle (*D. lardarius*) and the Peruvian hide beetle (*D. peruvianus*) will also attack leather (if not tanned and in good condition), skin, dead mice, rats, poison bait for mice, dead pigeons, or inactive beehives. They can also be attracted to large numbers of cluster flies inside of buildings under windows. 

A few species of moths are found inside buildings and are important pests, predominantly the webbing clothes moth (*Tineola bisselliella*), which is probably the most important pest on textiles, fur and feathers [25,26,60,61,62,63,64,65,66]. The webbing clothes moth is a pest on the textiles of animal wool (sheep or goat for example), fur, feathers, hair, felt, silk, carpets, rugs, blankets, upholstery, piano felts, fishmeal, milk powder, brush bristles, but often also come from dust [25]. The case bearing clothes moth (*Tinea pellionella*) has similar food requirements (wool, rugs, feather material, felts, hair, furs, but can feed also on spices, tobacco, hemp and skins) to the webbing clothes moth but is found less often in museums. It is said to need higher temperatures and more humid conditions. Other species occurring in museums are the brown-dotted clothes moth (*Niditinea fuscella*), the obvious moth (*Monopis obviella*) or the Indian mealmoth (*Plodia interpunctella;* not a pest on museum objects) [67].

All the keratine feeding insects can result in severe damage of insect collections, stuffed animals, and also textile museum objects. Not only museum objects can be a food source but also dead insects like flies accumulating under windows can attract *Anthrenus*, *Attagenus* or *Dermestes* species. Together with the webbing clothes moth, they are also found in dust where they find sufficient food. From these reservoirs of pests, new infestations can take place, making regular cleaning such an important part of modern IPM in museums.

### 2.3. Insect Pests on Plant Materials (Herbaria, Dried Food)

Some insects are feeding primarily on plant materials, for example the Anobiid biscuit beetle (*Stegobium paniceum*), cigarette beetle (*Lasioderma serricorne*), or spider beetles like the Australian spider beetle (*Ptinus tectus*), the golden spider beetle (*Niptus hololeucus*), the white marked spider beetle (*Ptinus fur*) or the hump-back spider beetle (*Gibbium psylloides*). The larvae can damage not only dried plants in herbaria collections and upholstery, but also textiles, mummies, animal mounts and insect collections. Biscuit beetles are known to eat objects with starch glue and also pictures or old grain mouse bait [68,69]. Cigarette beetles prefer dried plant materials (even tobacco) but can feed also on freeze-dried animal specimens. If stored food products like cereal, pasta, rice, dried fruits, seeds, dried fish, drugs, spices or bread are part of the collection or stored in the same place, they can be a source of the infestation. Biscuit beetles and cigarette beetles can especially cause severe damage to many objects in ethnographic collections because of their fast development and reproduction.

The two khapra/cabinet beetles (*Trogoderma angustum* and *Trogoderma germarium*) are also found inside buildings infesting herbaria, pumpkin vessels stained with liquids, dead mice, wasp nests, spider webs, and animal specimens. The Odd beetle (*Thylodrias contractus*), an important pest in the USA and also occurring in the UK, is seldom found in museums in central Europe but is also a pest in herbariums and other natural history collections (it was found recently in one museum in Bavaria).

All the species listed above prefer dried plant material as food, but are also able to infest keratine- or chitin-based materials (see Section 2.2.). In collections with mixed materials, they prefer feeding on the plants.

## 3. Insect Pests Found in Libraries, Archives and Paper

Some insects damaging materials can be found on historic books and paper. In historic libraries, the paper, book covers (bindings) using leather, parchment, cardboard, wood or wooden shelves can be infested by a few species of insect pests. Most important today is the biscuit beetle (*Stegobium paniceum*), the cigarette beetle (*Lasioderma serricorne*), the spider beetles like the Australian spider beetle (*Ptinus tectus*) or the white marked spider beetles (*Ptinus fur*)*.* The larvae of these pests are known to eat objects like the bookbindings with starch glue. Biscuit beetles can especially cause severe damage to historic books as they reproduce fast and spread quickly. In the past when historic libraries were still less climatised, the furniture beetle (*Anobium punctatum*) was also often found feeding on books, paper and bookbindings. Today, they are normally replaced by the biscuit beetles and sometimes the spider beetles, because these species are more tolerant towards humidity and temperature.

Another important group of pests on books and paper are the silverfish and book lice. Different species of silverfish are found inside of buildings (usually identified all as Silverfish): Silverfish (*Lepisma saccharina*), paperfisch (*Ctenolepisma longicaudatum*), firebrat (*Thermobia domestica*) and four-lined silverfish (*Ctenolepisma quadriseriata*) all feed mainly on detritus, mould, human skin or hair (textiles, cotton, silk), but can also damage paper, bookbindings, wallpaper, papier-mâché, starch glue and cellulosic materials. Book lice (Psocoptera) can also be found in high numbers if humid conditions present. Species like *Liposcelis* sp. usually eat mould and starch, but can damage paper, bookbinding’s, herbal specimens, wallpaper and even stuffed animals.

Both silverfish and book lice need high humidity and are found in buildings in high numbers only where humid conditions are found (60% RH or above). Regulating the climate and cleaning to reduce dust, microscopic fungi and other organic matter are two important methods to stop the last two group of pests. A few individuals occur in every building and should be tolerated in small numbers. For pests like biscuit beetles or cigarette beetles in a library, there is no level of tolerance and the infested objects need to be treated as soon as possible to prevent further spread and damage.

## 4. Insect Pests Found in Historic Buildings

Most of the species mentioned above can be also found in historic buildings. Furniture beetles like *Anobium punctatum* for example can damage structural timber, historic furniture on display or wooden floors. Because historic buildings are often found in park-like landscapes with some dead trees, the chance of an infestation from outside is higher then in museums inside of cities. Another source of infestation is untreated firewood that is stored inside of palaces or castles (for use or as display). Wood boring beetles needing higher wood moisture often find good conditions in historic buildings under the wooden floors or behind historic wood paneling, where temperature changes between the inside and the outside, can result in a humid microclimate.

Spider beetles, especially the humpback spider beetle (*Gibbium psylloides*), are often found in historic buildings where they live underneath floorboards feeding on plant materials (threshing waste) used as insulation. These insects are very tolerant to low temperatures and can even finish their life cycle at temperatures of 10 °C. They usually don’t damage any objects in the historic building but are annoying to visitors and the owners.

In historic buildings, fur beetles (*Attagenus*), carpet beetles (*Anthrenus*) or webbing clothes moth often have a resident population within the building. This can be underneath floorboards, but also unused chimneys, shafts or the attic. Here, dust, dead animals or animal nests can be a food source for many generations. Sealing a historic building to prevent insect entry poses a big challenge and many windows are not tight, resulting in the mass entry of cluster flies, for example.

All information on the food requirements and biology were collected from the homepage http://museumpests.net [19] (see pest fact sheets) and the literature [13,14,15,50,51,52,53,54]. Personal observations over the last 10 years in the field were also included (see also [58,60,61]).

## 5. Conclusions

Knowing the pest species and their biology is an important part of IPM in museums, libraries, archives and historic buildings. A lot of information is available in books and on the internet, as most species are also important pests for the food industry (the biscuit beetle, for example) and stores’ product protection (for example, the webbing clothes moths or tobacco beetle). Knowing the phenology and biology helps the search for infested objects, problems connected to the building, and being able to use this information against the pests. Regulating the humidity, for example, is the most efficient solution to stop the activity of furniture beetles, death watch beetles, most weevils feeding on wood, and also silverfish and related species. The results of the monitoring can also be used to argue for better cleaning and housekeeping, for example, if webbing clothes moths or carpet beetles are found in large numbers, even if only a few or no infested textile objects are present in the store or exhibition space.

Experience has shown in recent years, that normally pests are transported with infested objects into a collection [70,71], and that they rarely fly into the building through open windows or doors [62]. This is an important part of the prevention, and having a good record of what pests are present in the collection helps to notice such changes.

The most abundant pest species in housing collections are silverfish, webbing clothes moths, carpet beetles and biscuit beetles. All are common museum pest species feeding on animal fur and textiles made with animal fibers, feathers or felt. Only the biscuit beetles feed mainly on starchy materials like bookbinding’s. Dust and dead flies are an important food source for many pests and should be avoided. Large historic buildings are often difficult to seal and remain susceptible to infestations, which make long-term solutions costly and difficult to achieve.

New areas of IPM are required to better understand the spread of pests across Europe, as new species are being introduced from time to time across borders and museums where they were not present before. How this aspect is also related to climate change in the future will be an important field of research (see [72,73,74]).
